# Epigenetic Effects on Pediatric Traumatic Brain Injury Recovery (EETR): An Observational, Prospective, Longitudinal Concurrent Cohort Study Protocol

**DOI:** 10.3389/fneur.2020.00460

**Published:** 2020-06-12

**Authors:** Amery Treble-Barna, Jamie Patronick, Srivatsan Uchani, Noelle C. Marousis, Christina K. Zigler, Ericka L. Fink, Patrick M. Kochanek, Yvette P. Conley, Keith Owen Yeates

**Affiliations:** ^1^Department of Physical Medicine and Rehabilitation, University of Pittsburgh School of Medicine, Pittsburgh, PA, United States; ^2^Department of Population Health Sciences, Duke University School of Medicine, Durham, NC, United States; ^3^Safar Center for Resuscitation Research, Division of Pediatric Critical Care Medicine, UPMC Children's Hospital of Pittsburgh, Department of Critical Care and Pediatrics, University of Pittsburgh School of Medicine, Pittsburgh, PA, United States; ^4^Department of Health Promotion and Development, School of Nursing, University of Pittsburgh, Pittsburgh, PA, United States; ^5^Department of Psychology, Alberta Children's Hospital Research Institute, Hotchkiss Brain Institute, University of Calgary, Calgary, AB, Canada

**Keywords:** traumatic brain injury, precision medicine, childhood adversity, epigenetics, brain-derived neurotrophic factor

## Abstract

**Introduction:** Unexplained heterogeneity in outcomes following pediatric traumatic brain injury (TBI) is one of the most critical barriers to the development of effective prognostic tools and therapeutics. The addition of personal biological factors to our prediction models may account for a significant portion of unexplained variance and advance the field toward precision rehabilitation medicine. The overarching goal of the Epigenetic Effects on Pediatric Traumatic Brain Injury Recovery (EETR) study is to investigate an epigenetic biomarker involved in both childhood adversity and postinjury neuroplasticity to better understand heterogeneity in neurobehavioral outcomes following pediatric TBI. Our primary hypothesis is that childhood adversity will be associated with worse neurobehavioral recovery in part through an epigenetically mediated reduction in brain-derived neurotrophic factor (*BDNF*) expression in response to TBI.

**Methods and analysis:** EETR is an observational, prospective, longitudinal concurrent cohort study of children aged 3–18 years with either TBI (*n* = 200) or orthopedic injury (*n* = 100), recruited from the UPMC Children's Hospital of Pittsburgh. Participants complete study visits acutely and at 6 and 12 months postinjury. Blood and saliva biosamples are collected at all time points—and cerebrospinal fluid (CSF) when available acutely—for epigenetic and proteomic analysis of *BDNF*. Additional measures assess injury characteristics, pre- and postinjury child neurobehavioral functioning, childhood adversity, and potential covariates/confounders. Recruitment began in July 2017 and will occur for ~6 years, with data collection complete by mid-2023. Analyses will characterize *BDNF* DNA methylation and protein levels over the recovery period and investigate this novel biomarker as a potential biological mechanism underlying the known association between childhood adversity and worse neurobehavioral outcomes following pediatric TBI.

**Ethics and dissemination:** The study received ethics approval from the University of Pittsburgh Institutional Review Board. Participants and their parents provide informed consent/assent. Research findings will be disseminated via local and international conference presentations and manuscripts submitted to peer-reviewed journals.

**Trial Registration:** The study is registered with clinicaltrials.org (ClinicalTrials.gov Identifier: NCT04186429).

## Introduction

Traumatic brain injury (TBI) is a leading cause of morbidity and mortality in childhood (1). The scientific evidence available to guide prognosis, management, and treatment is disproportionately low relative to TBI's medical and societal burden. Children sustaining moderate to severe TBI often demonstrate neurobehavioral impairments that hamper their long-term functioning (2–4). Previous large-scale cohort studies in pediatric TBI have identified injury (e.g., severity, neuropathological, accidental vs. nonaccidental), developmental (e.g., age at injury, time since injury), child (e.g., premorbid functioning), and psychosocial (e.g., socioeconomic status, family functioning) factors as significant determinants of outcomes (5–12). Current prediction models including these factors, however, explain only ~35% of variance in outcomes (13). Without the identification of additional factors influencing recovery, this unexplained heterogeneity will remain one of the most critical barriers to accurate prognostication and to the development of evidence-based treatments for the neurobehavioral consequences of pediatric TBI (14, 15).

The addition of personal biological factors or “biomarkers” in our prediction models may account for a significant portion of unexplained variance. This would advance the field of pediatric TBI toward precision rehabilitation medicine, to thereby improve individual prognostication, predict response to rehabilitation, and identify novel targets for therapy development. A biomarker gaining traction in TBI research is genetic variation, with the hypothesis being that certain individuals may be genetically predisposed to worse recovery and outcomes (16–18). The investigation of genetics without consideration of factors that modulate gene expression, however, precludes a more integrative and comprehensive understanding of the biological and environmental mechanisms affecting recovery.

Over the last decade, the field of epigenetics has become central to studying the modulation of genetic phenotypes by environmental factors. Broadly defined, epigenetics involves biochemical processes that regulate gene expression without altering the corresponding primary DNA sequence (19). Through epigenetic processes, the social and biological environment of an individual impacts when and to what extent genes are expressed within each cell type. The best-characterized epigenetic mechanism is DNA methylation, with higher levels rendering the gene less transcriptionally active (20).

Among the strongest epigenetic influences on child development is the family environment, especially when it involves exposure to childhood adversity (21, 22). Children who experience greater adversity, such as poverty, separation from a parent, and maltreatment, show altered methylation profiles in genes involved in the function of the hypothalamic–pituitary–adrenal axis, immune response, and neuronal development and neuroplasticity (21, 23, 24). In turn, these altered methylation profiles are associated with higher prevalence of chronic health conditions (25), as well as a multitude of neurobehavioral phenotypes, including depression and anxiety, borderline personality disorder, attention problems, and poor impulse control (23, 24, 26–28).

In pediatric TBI, decades of research demonstrate worse outcomes in children exposed to greater adversity, including lower socioeconomic status, greater family dysfunction, and abusive head trauma as the injury mechanism (4, 5, 10, 29, 30). Despite these established findings, the biological mechanisms underlying these effects are unknown. Given the known influence of childhood adversity on both children's epigenetic profiles and recovery from pediatric TBI, the effect of childhood adversity on recovery may be mediated by epigenetic mechanisms.

The brain-derived neurotrophic factor (*BDNF*) gene is an ideal candidate for the first exploratory examination of epigenetic influences on neurobehavioral recovery following pediatric TBI because it is involved in both: (1) the biological response to childhood adversity and (2) neuroplasticity following TBI. BDNF is a well-studied member of the neurotrophin family of growth factors. It is released from neurons both pre- and postsynaptically and mediates apoptosis, neuronal differentiation, outgrowth of neurites, cell survival, and synaptic strengthening (31, 32). Thus, BDNF is intimately involved in brain development, neuroplasticity, and neuronal survival (33, 34), as well as complex cognitive processes, including learning/memory (35, 36) and executive functioning (37–40). A single-nucleotide polymorphism producing a valine-to-methionine substitution at codon 66 (val66met) in the *BDNF* gene is associated with reduced activity-dependent secretion of BDNF (41) and may modify methylation level at this position on the gene (42–44). In addition to other transcriptional and translational regulators (45, 46), *BDNF* gene expression, as is the case with many genes, is regulated in part by DNA methylation, such that higher methylation is associated with lower neuronal synthesis of BDNF (47).

*BDNF* DNA methylation epigenetically encodes the effects of childhood adversity on neurodevelopment. Multiple recent studies show that early adversity produces changes in *BDNF* methylation detectable in the brain and peripheral biosamples in both animal models (48–50) and humans (23, 51–53). Both higher *BDNF* methylation (23, 54) and lower BDNF protein levels (55–60) are associated with worse neurobehavioral outcomes in children with preterm birth, attention-deficit/hyperactivity disorder (ADHD), bipolar disorder, anorexia, and autism spectrum disorder.

The BDNF pathway is also involved in neural recovery from TBI. BDNF is upregulated in the frontal cortex and the hippocampus after experimental TBI and may mediate recovery processes after brain injury (61–63). In adult clinical studies, BDNF levels in cerebrospinal fluid (CSF) show a sharp spike within 24 h postinjury and steadily decline over 1 week (64, 65), providing evidence for BDNF upregulation and involvement in recovery post-TBI consistent with animal models. BDNF levels during the first week postinjury tend to be comparable in CSF (65) but lower in serum in adults with TBI relative to nonbrain-injured controls (65, 66). Furthermore, acute and chronic BDNF levels in CSF and serum/plasma after TBI in adults predict outcomes, including mortality (65), global outcomes (66, 67), and long-term functional cognition and depression (68, 69). Far fewer studies have examined BDNF levels after pediatric TBI. Initial evidence shows a sharp peak in BDNF levels immediately following injury, followed by subsequent declines (70, 71), similar to that shown in adults. Higher CSF and plasma/serum BDNF levels predicted better global recovery in pediatric TBI studies (71, 72), but to our knowledge, no known studies have examined *BDNF* genetics or epigenetics in association with TBI outcomes in children.

**Figure 1:**
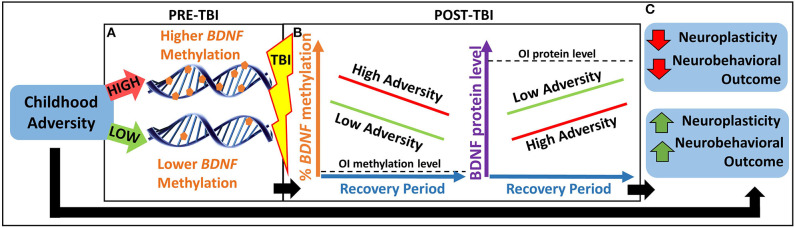
Heuristic model of our primary hypothesis. **(A)** Children with greater childhood adversity may have higher pre-TBI *BDNF* methylation, which may lead to **(B)** higher *BDNF* methylation and lower BDNF protein levels in response to TBI and over the recovery period, resulting in lower neuroplasticity and worse neurobehavioral outcomes. **(C)** This pathway may contribute to a biological explanation for the association between greater childhood adversity and worse neurobehavioral outcomes following pediatric TBI. BDNF, brain-derived neurotrophic factor; OI, orthopedic injury; TBI, traumatic brain injury.

Thus, the overarching goal of our study is to investigate an epigenetic biomarker involved in both childhood adversity and postinjury neuroplasticity to better understand heterogeneity in neurobehavioral outcomes following TBI. Our primary hypothesis is that childhood adversity will be associated with worse neurobehavioral recovery in part through an epigenetically mediated reduction in *BDNF* expression in response to TBI (see Figure 1).

The study uses an observational, prospective, longitudinal concurrent cohort design to examine the epigenetic influence of the biologically relevant *BDNF* pathway on neurobehavioral recovery in 200 children with nonpenetrating complicated mild to severe TBI relative to 100 children with orthopedic injury (OI) but no brain injury at three time points (acute, 6 months, 12 months) during the first year of recovery. As one of the first studies of epigenetic effects in pediatric TBI, this is an exploratory pilot study. The study will establish a longitudinal biorepository that is innovative in its integration of acute and chronic biological samples with comprehensive data characterizing preinjury childhood adversity and postinjury neurobehavioral functioning. Only a portion of each biosample will be used for the analyses proposed in the present study; the remaining biosamples will be stored in the biorepository. This rich biorepository will be expanded longitudinally in subsequent studies and will provide opportunities to evaluate other epigenetic, genetic, and protein biomarkers and biological mechanisms in future secondary analyses. The results of the present pilot study will guide the design of a subsequent larger multisite study that will be sufficiently powered to examine additional epigenetic biomarkers while controlling for additional sources of heterogeneity in outcomes.

Our specific aims and hypotheses are as follows:

Aim 1: Examine differences between TBI and OI in *BDNF* methylation and BDNF protein levels over the recovery period. We hypothesize that *BDNF* methylation will be higher, and BDNF protein levels will be lower, in children with TBI relative to OI at all time points, and that *BDNF* methylation will decrease, and BDNF protein levels will increase, in the TBI group over time but will remain relatively stable in the OI group.
Aim 2: Characterize trajectory classes of *BDNF* methylation and BDNF protein levels following TBI while controlling for potential demographic, injury, and lifestyle covariates. We hypothesize the presence of at least two *BDNF* methylation trajectory classes and two BDNF protein level trajectory classes representing varying degrees of recovery from TBI. We will explore age, sex, pubertal status, TBI severity, overall injury severity, body mass index (BMI), and smoking as potential covariates.
Aim 3: Test the indirect effect of childhood adversity on neurobehavioral recovery following pediatric TBI, as mediated by *BDNF* methylation and BDNF protein level trajectory classes.

## Methods and Analysis

### Study Overview

Children aged 3–18 years with either complicated mild to severe nonpenetrating TBI (*n* = 200) or OI (*n* = 100) are recruited from consecutive overnight admissions to the UPMC Children's Hospital of Pittsburgh (CHP). The sample size of 200 children with TBI and 100 children with OI was chosen based on two considerations: (1) feasibility of recruitment over the study period based on historic numbers of children with TBI meeting eligibility criteria at our site and (2) adequacy of statistical power for proposed analyses taking into consideration participant attrition over the study period (see section Statistical Methods and Power). We selected the age and severity ranges to optimize sample size for this initial exploratory pilot study and to examine the effects of demographic and injury factors as potential covariates to inform design of future studies. In addition, most neurobehavioral measures selected for use in the study have age-specific forms spanning this age range. To ensure an adequate distribution across TBI severity and because disproportionately more children sustain complicated mild relative to moderate to severe TBI, we will stratify TBI recruitment into two severity groups (complicated mild vs. moderate/severe) and recruit consecutively within each group until we have at least 20% of the total sample in the moderate to severe TBI group. Children with OI were selected as a comparison group to control for premorbid risk factors for injury of any kind and to equate for the experience of having a traumatic injury and associated medical treatment (73, 74). In addition, comparing biomarkers between traumatically injured children with and without injury to the brain will help differentiate general injury effects on systemic biomarkers from those that are specific to brain injury (75). Children with spinal cord injury are excluded from both the TBI and OI groups to avoid confounding from neuroinflammation and brain involvement.

Data are collected at three time points postinjury. Acute data collection occurs before hospital discharge, and chronic data collection occurs at 6 and 12 months postinjury during outpatient follow-up visits to CHP. Data are collected pertaining to hypothesized predictors of neurobehavioral recovery (e.g., demographics, injury information, child premorbid functioning, childhood adversity, and biomarkers), neurobehavioral outcomes (e.g., neuropsychological test performance, parent-rated neuropsychological, emotional/behavioral, and adaptive functioning), and potential covariates/confounders (e.g., BMI, pubertal status, tobacco/nicotine exposure, bodily injury severity). Recruitment began in July 2017 and will occur for ~6 years, with data collection complete by mid-2023.

DNA methylation patterns are tissue specific and reflect the local environment of each cell type, and examination of brain tissue is not feasible in patients who survive their injuries; therefore, determining which types of biosamples serve as effective proxies for the CNS environment is an important methodological consideration. Moreover, to be useful as a clinical biomarker, markers must be found in readily accessible biosamples, such as blood or saliva. Several previous studies have shown that methylation in peripheral biosamples, including blood and saliva, is strongly correlated with methylation in brain tissue or CSF (46, 76–81) for several target genes, including *BDNF* (46, 77). Methylation measured in peripheral biosamples may be of even greater prognostic value in TBI compared to neuropsychiatric conditions because brain-specific biomarkers may enter blood circulation in TBI not only through disruption to the blood–brain barrier but also through the glymphatic system (82). Finally, a recent review of the “tissue issue” concluded that the limitations of examining epigenetic brain biomarkers in peripheral tissues or biosamples are surmountable and discussed methodological advantages of using peripheral biosamples: serial biosampling, longitudinal study designs and related causal modeling options, large participant sample sizes yielding statistical power to detect modest effects, and translational utility in clinical settings (83).

In light of these methodological considerations, we collect saliva and blood biosamples at acute and chronic time points in all participants. We also collect acute serial CSF samples in the small subsample of participants with extraventricular drain (EVD) placement to manage intracranial pressure as part of guidelines based standard of care (84).

### Inclusion Criteria

Children in both groups must be from 3 to 18 years of age at the time of the injury. Children with TBI must be hospitalized overnight for a complicated mild to severe nonpenetrating TBI as defined by the lowest postresuscitation Glasgow Coma Scale (GCS) score (85). Complicated mild TBI is defined as a GCS of 13–15 with neuroimaging indicating intracranial or parenchymal injury or depressed/displaced skull fracture. Moderate TBI is defined as GCS of 9–12. Severe TBI is defined as GCS of 3–8. Children are included in the OI group if they sustain a bone fracture, excluding to the skull or face, without any signs of head trauma or brain injury (e.g., nausea/vomiting, headache, loss of consciousness, GCS below 15 at any point).

### Exclusion Criteria

Children in both the TBI and OI groups meet the following exclusion criteria: (a) non-English-speaking child or non-English-speaking parents/guardians; (b) documented or parent-reported history of previous TBI/concussion requiring overnight hospitalization; (c) preinjury neurological disorder or intellectual disability; (d) preinjury psychiatric disorder requiring hospitalization; (e) spinal cord injury; (f) sensory or motor impairment precluding study measure completion; and (g) pregnancy at the time of study participation. Participants are also excluded if at least one biosample is not able to be collected within 7 days of the injury. Children in the TBI group are not excluded if they also have an OI.

### Study Procedures

The study staff screen the CHP electronic medical record (EMR) daily to identify children who are potentially eligible for study participation. Children are housed in one of two settings when they are identified: (1) the acute care trauma unit or (2) the pediatric intensive care unit (PICU). Children housed in the acute care trauma unit within the first week of their injury are approached about the study. The study staff approach families and conduct brief interviews about the child's preinjury functioning and medical history to confirm eligibility and obtain informed consent prior to any data collection. Interested parent(s) of minor children, and 18-year-old patients capable of consenting on their own behalf, provide written informed consent. Children over the age of 8 capable of understanding study procedures provide assent.

Children with more severe injuries are sometimes housed in the PICU during the acute biosample window (i.e., 1 week postinjury). If a potentially eligible child is in the PICU, the bedside nurse collects the acute blood sample at the same time as a clinical blood draw, before consent has been obtained per an Institutional Review Board (IRB)-approved waiver of informed consent. In addition, if a child with severe TBI has an EVD placed as part of routine clinical care, the bedside nurse collects daily CSF samples up to 1 week postinjury prior to study consent. Families of children whose samples are collected preconsent are approached about study participation once the child is medically stable, usually after discharge to the acute care trauma unit. If the family declines to participate or the child is discovered through screening questions to be ineligible, the preconsent samples are destroyed. Destruction of preconsent samples is requested by the research coordinator and completed by research nurses in the CHP Pediatric Clinical and Translational Research Center (PCTRC). Documentation of sample destruction is maintained both by the PCTRC and by the research coordinator.

For children and families who consent to study participation, an additional postconsent blood sample is obtained by the bedside nurse in addition to any preconsent acute blood and CSF samples. The study staff collect a saliva sample from the child. Prior to discharge, parents complete measures pertaining to demographic information, premorbid child functioning, childhood adversity, child resilience, and the child's pre- and postnatal exposure to nicotine/tobacco. Children 8 years and older complete a self-report measure of puberty status, and children 10 years and older also complete a self-report measure of tobacco/nicotine use. Injury information (e.g., mechanism, neuroimaging results, GCS) and BMI are obtained from the child's EMR.

The study staff contact families to schedule their 6- and 12-month follow-up research visits at CHP. Follow-up visits last ~90 min. At each visit, PCTRC research nurses collect blood via venipuncture, and the study staff collect saliva. Parents complete the same measures as the acute visit, this time to assess postinjury functioning (see Table 1 and Section Measures). Children complete the National Institutes of Health Toolbox-Cognition Battery (NIHTB-CB) and self-report measures of puberty and tobacco/nicotine use. Families who refuse to return for follow-up research visits are given the option to return parent study measures and the child saliva sample via mail. Families are provided with instructions for how to collect the saliva sample and complete the parent measures as well as return packaging and postage.

**Table 1:** Study measures organized by domain, source of data, and assessment occasion.

**Domain/measures**	**Source/reporter**	**Parent/child time to complete (min)**	**Acute (preconsent)**	**Acute (postconsent)**	**6 months**	**12 months**
**BIOMARKER COLLECTION**						
Blood sample	C	10	X	X	X	X
Saliva sample	C	5–10		X	X	X
CSF sample	C	N/A	X			
Injury information	EMR	N/A	X			
Demographics	P	15		X	X	X
**CHILD FUNCTION**						
BRIEF-2	P	10		X^†^	X	X
SDQ	P	10–15		X^†^	X	X
Vineland-3	P	10–15		X^†^	X	X
NIHTB-CB	C	30–45		X	X	X
**CHILDHOOD ADVERSITY**						
PAT	P	5–10		X	X	X
CHIP	P	10–15		X^†^	X	X
**POTENTIAL COVARIATES/CONFOUNDERS**						
Pubertal status self-report	C	5		X	X	X
Tobacco/nicotine self-report	C	5		X	X	X
Caregiver tobacco/nicotine screener	P	5		X	X	X

† *Retrospective ratings by parent at acute assessment to assess premorbid functioning*.

*BRIEF-2, Behavior Rating Inventory of Executive Function, Second Edition; C, child; CHIP, Child Health and Illness Profile; CSF, cerebrospinal fluid; EMR, electronic medical record; NIHTB-CB, NIH Toolbox-Cognition Battery; P, parent; PAT, psychosocial assessment tool*.

Families are compensated for their time at the acute and follow-up visits and reimbursed for parking and transportation costs. Multiple methods are used to track and contact participants and reduce attrition.

### Measures

Table 1 provides an overview of study measures organized by domain, source of data, and assessment occasion.

### Hypothesized Predictors

#### Demographics

Child and family demographic information is collected at both the acute time point and at the 6- and 12-month follow-ups. Parents answer questions about the child and family members' age, sex, race, ethnicity, and relationship to the child (e.g., biological parent, legal guardian, etc.), as well as socioeconomic status (e.g., family income, parent education level, parent employment, use of financial assistance programs), household composition, and marital status.

#### Injury Information

The study staff extract information about the characteristics of the child's injury from their EMR. Specifically, the child's initial, postresuscitation, and best in 24-h GCS scores, MRI or CT findings, date and time of injury, and mechanism of injury are noted.

#### Preinjury Child Function

At the acute visit, parents report on their child's preinjury functioning through retrospective recall. The efficacy of this method has been demonstrated in previous studies (86, 87).

To assess everyday executive functioning, parents complete the Behavior Rating Inventory of Executive Function, Second Edition (BRIEF-2) or Preschool Version (BRIEF-P) (88, 89). Three composite scores are computed for behavioral regulation, emotion regulation, and cognitive regulation, as well as a global executive composite.

The Strengths and Difficulties Questionnaire (SDQ) measures psychological adjustment. Subscales include Emotional Symptoms, Conduct Problems, Hyperactivity–Inattention, Peer Problems, and Prosocial Behavior. A Total Difficulties score is also provided. Four different versions are administered based on the child's age.

Adaptive functioning is measured using the Vineland Adaptive Behavior Scales, Third Edition (Vineland-3). Parents complete items designed to assess their child's ability to perform day-to-day activities in the domains of Communication, Daily Living, and Socialization. Composite scores are computed for each domain, as well as a general Adaptive Behavior Composite (90).

#### Childhood Adversity

Parents complete the Psychosocial Assessment Tool (PAT) as a measure of psychosocial risk and childhood adversity, particularly in the context of the family in a pediatric setting. The PAT evaluates a variety of domains: family structure and resources, social support, patient, sibling, and caregiver problems (internalizing, externalizing, social, cognitive), family beliefs, and caregiver stress reactions. Subscale scores are calculated for each of these domains, as well as a total PAT score (91).

Resilience may buffer the effects of childhood adversity (92) and has recently gained interest in predicting outcomes following TBI (93, 94). Parents complete the Resilience items from the Child Health and Illness Profile (CHIP), child edition (6–11 years; 19 items) or adolescent edition (12–18 years; 38 items) (95, 96). The Resilience domain measures the parent's assessment of the child's family involvement, social problem solving, and physical activity.

### Biomarker Collection and Processing

CSF is collected by the bedside nurse into a CSF vial and refrigerated until processing within 48 h by the CHP PCTRC. Processing involves separation and aliquoting of supernatant for future protein level analysis and the cell pellet for genetic and epigenetic analysis. Processed samples are stored at −80°C.

Acute blood samples are collected by the child's bedside nurse from his or her IV if patent or via venipuncture with parent and child permission. Chronic blood samples are collected via venipuncture by a PCTRC nurse. Up to 10 cc of blood is collected at each draw into ethylenediaminetetraacetic acid (EDTA) vacuum tubes and refrigerated until processing within 48 h by the PCTRC. Processing involves separation and aliquoting of plasma for future protein level analysis and the buffy coat (leukocytes) for DNA extraction using a simple salting protocol for genetic and epigenetic analysis. Processed samples are stored at −80°C.

Up to 2 ml of saliva is collected by the study staff using Oragene DNA sample collection kits (DNA Genotek, Ontario, Canada). To collect saliva, the child spits into the Oragene collection tube for 2–5 min until liquid saliva reaches the fill line. If the child is unconscious or unable to spit, study staff instead use a sponge to gently swab the child's gums and inner cheeks and then wring the saliva out of the sponge into the collection tube until the fill line is reached. The study staff then close the tube's lid to release the stabilizing liquid and store the sample at ambient temperature until processing. DNA is extracted using the reagents and protocol from the manufacturer.

### Outcomes

Child neuropsychological function is assessed acutely and at each follow-up visit using the NIHTB-CB (97). The NIHTB-CB is a 30-min battery of standardized neuropsychological tests administered on an iPad. The NIHTB-CB provides norm-referenced scores for the domains of language, episodic memory, processing speed, working memory, and executive function, as well as an overall cognitive function composite score.

Parent-reported child postinjury executive, emotional/ behavioral, and adaptive functioning are measured at follow-up visits using the BRIEF-2/P, SDQ, Vineland-3, and resilience items from the CHIP. Postinjury adversity is measured using the PAT and child resilience by the CHIP.

### Potential Covariates/Confounds

*BDNF* methylation and protein levels vary by age, sex, and pubertal status (98–101), as well as lifestyle factors including BMI and smoking (101–103); therefore, we are collecting information on these factors at all time points to be investigated as potential covariates. We will also examine the effect of overall bodily injury severity because polytrauma can be a significant confounder in biomarker studies (75, 104).

Pubertal status is measured for children ≥8 years using the self-report Pubertal Development Scale (105). The scale provides a Pubertal Development Score, which shows adequate agreement with direct clinical assessment of Tanner staging (106).

The child's BMI is obtained at each visit from the EMR or study staff.

Parents are given a brief screener measuring the child's exposure to nicotine and tobacco. The screener measures maternal use and secondhand exposure during pregnancy, as well as the child's secondhand exposure. Children 10 and up also complete a brief self-report measure about their own nicotine and tobacco use. They are asked how frequently they have used a variety of nicotine/tobacco products. Children complete the questionnaire on paper while their parents are out of the room and are informed that their responses will be kept private.

The Injury Severity Score (ISS) (107) measures overall bodily injury severity and is obtained from the local trauma registry. The score for injury to the head will be excluded to avoid confounding with TBI severity.

### Data Management

All electronic and paper data storage is IRB and HIPAA compliant through separation of identifiable and deidentified data, storage of paper data, and samples in locked cabinets in locked offices, use of secure servers, and encrypted and password-protected files and computers. Research Electronic Data Capture (REDCap) is used for electronic data storage. REDCap is a web-based platform that allows a common database for data entry and rules for data handling. All data that are entered into REDCap are checked for discrepancies using double verification and error checks.

### Data Reduction, Missing Data, and Attrition

Measures were selected that provide composites or summary indices representing each construct, reducing the need for substantial data reduction. Children will be eligible to remain in the study if at least one acute biosample (CSF, blood, or saliva) is obtained and both primary parent questionnaires (BRIEF-2/P, SDQ) are completed. This will reduce missing data on key variables and retain participants who refuse blood collection or are unable to produce sufficient saliva. In addition, statistical techniques such as mixed models, latent class trajectory analysis, and path analysis will be used, which estimate models based on all available data using maximum likelihood estimation (108). Consistent with previous studies in this population, we conservatively estimate that ~75% of participants will complete the follow-up visits (150 TBI; 75 OI) (2, 4, 5). Our power analyses account for this reduced participant sample size at follow-up.

### Data Analyses

#### Biomarker Analysis

DNA extracted from saliva, blood, and CSF will be processed for methylation analysis. We currently plan to use EpiTect Methyl II PCR assays (Qiagen, Germantown, MD) to assess targeted methylation sites in and around the *BDNF* gene; however, due to rapid changes in technology for this type of data collection, when data collection is to begin, we will evaluate whether this method remains our best choice. We will target a select number of *BDNF* CpG islands for this initial study that showed the strongest correlation between blood and CSF in our preliminary data (109) and show significant correlations between *BDNF* methylation in blood and postmortem prefrontal cortex brain tissue according to the searchable online Blood Brain DNA Methylation Comparison Tool (110). Additional *BDNF* methylation targets may be examined in secondary analyses. The resulting data will estimate the methylation level at each CpG site by beta values (i.e., percent methylation). Beta values range from zero to 1, with zero indicating a null methylated site and 1 indicating a fully methylated site. *M* values will then be calculated and used in statistical analysis. (111, 112) Plasma BDNF protein levels will be measured using an ELISA kit according to the manufacturer's instructions. BDNF ELISA kits are available from several manufacturers (e.g., RayBiotech, R&D Systems). We will select a manufacturer at the time of analysis based on available specifications. For quality control, biosamples will be batch analyzed, assays will include biological duplicates and technical replicates, and data will be evaluated by two individuals blinded to phenotype.

Before our main analyses, we will examine the concordance of *BDNF* methylation across saliva, blood, and CSF and compare the predictive value of epigenetic results across biosample types. Based on these results, we may determine that saliva or blood biosamples are more informative than the other, and these results will guide which biosample(s) are used for measurement of *BDNF* methylation in our primary analyses.

#### Statistical Methods and Power

##### Aim 1: Examine differences between TBI and OI in BDNF methylation and BDNF protein levels over the recovery period

Unadjusted *BDNF* methylation and protein levels will be compared between TBI and OI groups using mixed effects analysis of variance (ANOVA; time as within effect; group as between effect). Mixed effects ANOVA allows for the modeling of effects on continuous outcomes (BDNF methylation and protein levels), is flexible with missing data, and provides explicit estimation of between group and within group variance. We will use appropriate transformations if normality is violated. We expect a significant group × time interaction and group differences at each time point. Alpha will be set at 0.05, and we will adjust for multiple comparisons using Bonferroni corrections. We expect sufficient power (≥0.8) to detect even a small interaction effect (partial η^2^ = 0.01) assuming correlation of 0.5 between repeated measures (G^*^Power 3.1.9.2).

##### Aim 2: Characterize trajectory classes of BDNF methylation and BDNF protein levels following TBI while controlling for potential demographic, injury, and lifestyle covariates

We will use latent class trajectory analysis (LCTA) (113) of *BDNF* methylation and protein levels separately, with the goal of identifying clusters of children with TBI who follow similar recovery trajectories. Some of the major benefits of LCTA are identification of distinct classes, simplification of complex data, and identification of adverse trajectories for potential intervention targets. Another strength given our heterogeneous sample is that probability of membership in each latent class is identified based on biomarker values over time as well as scores on significant covariates. Covariates to be tested will include age, sex, pubertal status, TBI severity, overall injury severity, BMI, and smoking. We will examine biomarker distributions to determine the need for transformations (114). Model selection will involve the iterative estimation of the number and shape of trajectory classes and identification of the best fitting model utilizing the following verified fit indices: Bayesian information criterion statistic, model estimation convergence, percentage of population in each subgroup (>10%), minimization of the residual variance statistic, and examination of posterior subgroup classification probabilities (115–117). Although the literature on power analyses for LCTA is limited (117), the power to identify the appropriate number of classes is highly dependent on separation of the trajectories (115). We believe our final sample is sufficient for several reasons: (1) we have a strong theoretical basis for our hypothesis of at least two classes and expect the classes to be relatively distinct; (2) we intend to evaluate different forms of the extracted model using verified fit indices (115); and (3) LCTA with covariates has been successfully applied in smaller TBI samples, *n* = 100–138 (75, 118–120).

##### Aim 3: Test the indirect effect of childhood adversity on neurobehavioral recovery following pediatric TBI, as mediated by BDNF methylation and BDNF protein level trajectory classes

**Figure 2:**
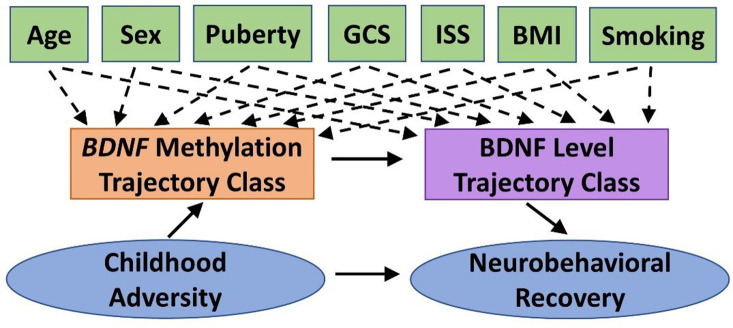
Theoretical model guiding Aim 3 statistical analyses. We will test the indirect effect of childhood adversity on neurobehavioral recovery following pediatric traumatic brain injury (TBI), as mediated by *BDNF* methylation and BDNF protein level trajectory classes while adjusting for potential covariates and confounders. BDNF, brain-derived neurotrophic factor; BMI, body mass index; GCS, Glasgow Coma Scale; ISS, Injury Severity Score.

We will conduct path analysis to test the theoretical model shown in Figure 2 within the TBI group. The theoretical model represents the relation among constructs rather than specific measures to allow for fluidity in our approach. For example, we will examine this model for each of the four primary measures of neurobehavioral outcome (NIHTB-CB, BRIEF-2/P, SDQ, Vineland-3). The specific biosamples used for *BDNF* methylation will be informed by preliminary analyses (see *Biomarker Analysis*). Path analysis enables a graphical representation of complex relationships, can explore direct and indirect effects among the variables (e.g., mediation), and infer causal directionality between effects. *BDNF* methylation and protein level trajectory class adjusted for covariates (Aim 2) will be tested as categorical mediators. Neurobehavioral recovery on each outcome will be modeled controlling for premorbid functioning (with the exception of NIHTB-CB). We estimated sample size requirements to adequately power the three single mediator models that would comprise our double-mediator model, as current guidelines on sample size for double mediator models are limited. The power of a single mediator model depends on the effect of the independent variable (IV) on the mediator and the effect of the mediator on the DV, controlling for the IV. Using Cohen's effect size criteria, we expect to have sufficient power (≥0.8) if both effects are at least “halfway” (between small and medium effects) (121). However, we would only need 115 participants if one effect is “halfway” and the other is medium. Thus, our final sample is conservative.

## Ethics and Dissemination

The study received ethics approval from the University of Pittsburgh Institutional Review Board, STUDY19040402. No significant risks are associated with participation in the study, and participation does not affect the care provided to participants.

The waiver of informed consent does not adversely affect the rights and welfare of the research participants because the participants are already undergoing blood draws for clinical care and the additional research blood draw alongside the clinical blood draw does not increase risk to the participant. In addition, the collection of CSF from the EVD does not impose any risk to the participant, and CSF samples would otherwise be discarded. Finally, the waiver does not adversely affect the rights and welfare of the research participants because consent will be obtained from the parent/child prior to drawing additional samples and the preconsent samples are destroyed if the parent/child does not consent to the study.

The study procedures are associated with a minimal risk of parent or child psychological discomfort. Parents and children are only approached with the bedside nurse's approval once the child is medically stable. The study staff approach families sensitively and respectfully to minimize any discomfort associated with the study and terminate all procedures immediately upon request or if the child or parents are experiencing any significant overt discomfort.

Although families do not receive any direct benefit for their participation, they do receive several incentives at each time point. Parents receive a letter summarizing their child's results on the NIHTB-CB along with the offer of referral for outpatient neuropsychological evaluation if indicated. All families of children with TBI receive brochures about CHP clinical neuropsychology services and other local brain injury resources.

Personal biomarker results will not be disclosed to research participants for several reasons: (1) the biological samples are being analyzed solely in the context of a research study; (2) the research findings, if provided, may be clinically irrelevant to available strategies for treatment; and (3) the research laboratory is not Clinical Laboratory Improvement Amendments (CLIA) certified to provide data upon which to base subsequent clinical decisions. The consent form describes that additional biomarkers may be examined in participant biosamples in the future.

The study staff are trained to ensure that data are secure and confidentiality is maintained at all times. All staff sign a confidentiality agreement and are only given the minimum access to data and patient information necessary to perform their duties. All identifiable data are kept in a secure and locked private office and is accessible only to study staff. Biological samples and all study forms are labeled with nonidentifying ID codes, and any links between the ID codes and identifiers are stored separately. Any data that are exported from the secure REDCap database is deidentified prior to export unless the identifier is necessary for analysis.

Research findings will be disseminated via local and international conference presentations and manuscripts submitted to peer-reviewed journals.

## Discussion

### Strengths and Potential Limitations

Our study has several strengths and potential limitations. In terms of strengths, the epigenetic focus, establishment of a longitudinal biorepository, and administration of the PAT and NIHTB-CB are, to our knowledge, novel contributions relative to previous large-scale cohort studies in pediatric TBI. The study of an epigenetic biomarker integrates the effects of genetics and the child's environmental context on recovery, providing a potentially powerful explanation of heterogeneity in neurobehavioral outcomes following pediatric TBI. Comparing biomarkers between traumatically injured children with and without injury to the brain will help differentiate general injury effects on systemic biomarkers from those that are specific to brain injury. The biorepository established for this study is innovative in its integration of acute and chronic biological samples with comprehensive data characterizing preinjury childhood adversity and postinjury neurobehavioral functioning and can be built upon longitudinally in subsequent studies, providing opportunities for the evaluation of many other epigenetic, genetic, and protein biomarkers and biological mechanisms contributing to recovery from pediatric TBI.

Regarding potential weaknesses, the wide age range and breadth of TBI severity of participants introduces greater heterogeneity than more restrictive age and severity inclusion criteria. Given that this is a single-site exploratory pilot study, however, we chose to be more inclusive to ensure a sufficient sample size for the proposed analyses. The effects of demographic and injury factors will be explored as potential covariates to inform the design of future studies. Similarly, *BDNF* methylation can be affected by other factors that are not measured in our study, including alcohol and drug exposure, diet, and exercise. We would look to consider these factors in future larger studies. Whether methylation in peripheral biosamples provides meaningful information regarding methylation in the brain is an inherent challenge to epigenetic research and remains to be further elucidated. Should the targets examined fail to show correlation of *BDNF* methylation between peripheral biosamples (blood and saliva) and CSF or fail to show associations with childhood adversity and neurobehavioral outcomes, the establishment of the biorepository in this study will allow for many additional epigenetic biomarkers to be explored. Finally, we acknowledge that *BDNF* is only one of many epigenetic pathways potentially influencing recovery. Biomarkers of interest to our research questions and available for subsequent analyses will include additional *BDNF* methylation targets, *BDNF*, neurotransmitter (17, 122) and inflammatory (123) genotypes, and additional biomarkers related to neuroplasticity and inflammation, given their role in both childhood adversity and recovery from TBI.

### Significance

Successful completion of the proposed aims has the potential to improve patient outcomes by advancing the field of pediatric TBI toward precision rehabilitation medicine. The unexplained heterogeneity in outcomes that plagues the field of TBI suggests a critical need to harness the potential of personal biology in predicting individual patient outcomes and response to interventions. The measurement of *BDNF* methylation and levels from accessible biosamples may serve as an early postinjury biomarker that more accurately predicts neurobehavioral outcomes following pediatric TBI, thereby allowing for earlier identification of children at greatest risk for poor recovery and the early provision of targeted intervention. Further understanding of the role of *BDNF* in recovery from TBI and the preinjury environmental determinants of its expression may bring us closer to being able to translate interventions to humans that have increased *BDNF* expression and improved cognitive outcomes in animal models, namely, environmental enrichment (63, 124) and exercise (125, 126). Finally, no neuroprotective therapies are currently available for TBI (127). Thus, demonstrating a possible role for *BDNF* epigenetics in mediating neurobehavioral outcomes will identify *BDNF* methylation as a possible target for the development of medical therapeutics. Interventions using epigenetic-based therapies have shown promise in experimental models of TBI (128, 129). This work will provide the foundation to develop biologically grounded, personalized approaches to treatment and rehabilitation that may apply across multiple child and adult brain disorders.

## Author Contributions

AT-B, KY, and YC designed the study with input from EF, PK, and CZ. AT-B and YC developed the biosampling protocol and analysis plan. AT-B and CZ developed the biostatistical plan. AT-B and JP wrote the first draft of the manuscript. JP, SU, and NM collected data and contributed to procedure improvements and clinical research coordination. All authors read, critically revised, and approved the final version of the manuscript.

## Conflict of Interest

The authors declare that the research was conducted in the absence of any commercial or financial relationships that could be construed as a potential conflict of interest.
